# A finite element analysis and cyclic load experiment on an additional transcortical-type hole formed around the proximal femoral nail system’s distal locking screw

**DOI:** 10.1186/s12891-022-05006-4

**Published:** 2022-01-27

**Authors:** Hong Man Cho, Seung Min Choi, Ji Yeon Park, Young Lee, Jung Hyung Bae

**Affiliations:** 1 Department of Orthopedic Surgery, Gwangju Veterans Hospital, 99 Cheomdanwolbong-ro, Gwangsan-gu, Gwangju, 62284 South Korea; 2 Department of Orthopedic Surgery, Inchon Veterans Hospital, Inchon, South Korea; 3 Veterans Medical Research Institute, Veterans Health Service Medical Center, Seoul, South Korea; 4 ENS Corp, Bio-medical Material and Component Service Center, Gwangju, South Korea

**Keywords:** Intertrochanter fracture, Femur, Hole, Distal screw, Proximal femur nail, Complication

## Abstract

**Background:**

A complication associated with the distal locking screw used in the proximal femoral nail (PFN) system is the formation of accidental additional holes. We hypothesized that an increase in stress around additional holes is a relevant factor contributing to fractures. This study aimed to evaluate stress changes in the cortical bone around additional screw holes using finite element analysis.

**Methods:**

Proximal femoral nail antirotation (PFNA)-II (Synthes, Solothurn, Switzerland) was inserted into a femur model. An additional 4.9-mm transcortical hole was made either anteriorly (anterior hole model) or posteriorly (posterior hole model) to the distal locking screw. Finite element analysis was used to calculate compression, tension, and load limits to investigate stress around additional holes with respect to the direction of screw penetration and degree of osteoporosis. The results were then compared with those of mechanical testing. A 31A-21 type intertrochanteric fracture was applied. As a control group, a model without additional holes (no-hole model) was developed. Repeated load-loading tests were performed on 10 model bones per model group.

**Results:**

Tensile stress was significantly greater in the no-hole model when additional screw holes were present, and the anterior hole showed a higher maximum stress value than the posterior hole, suggesting that the anterior hole was more susceptible to fracture. The change in tensile stress first appeared in the hole around the lateral cortical bone and proceeded to the medial side. Biomechanical testing showed that fractures around the distal locking screw occurred in 0 cases of the no-hole, 10 of the anterior hole, and 9 of the posterior hole models.

**Conclusions:**

During PFN surgery for intertrochanteric fracture, holes with distal locking screws fixed and removed at the anterior and posterior of the nail can be a risk factor for fractures in the surrounding area.

## Background

The use of distal locking screws in the proximal femoral nail (PFN) system surgically stabilize femoral intertrochanteric fractures, provide length and rotational stability, and restrain the movement of the distal tip of the nail in broad medullary canals [1]. Although improvements in the PFN system have been made, complications due to the distal locking screw still occur [2]. One of the complications associated with the distal locking screw is the incorrect placement of the target device, resulting in the creation of additional holes into which the distal locking screw may be inserted. When the orthopedic surgeon finds this screw malposition, the screw is removed and reinserted in the correct location. However, it is difficult to predict the risk of future fractures associated with these additional holes after screw removal. Therefore, to determine the risk of fracture around additional screw holes, this study aimed to investigate the stresses formed around these holes with respect to the direction of screw penetration and the degree of osteoporosis. To this end, the authors used finite element analysis (FEA) to calculate the compression, tension, and load limits around additional transcortical-type screw holes and compared the results to those of mechanical tests using the saw bones of intertrochanteric fracture models. Today, computer-aided finite element analysis (FEA) was used to solve processes such as metal turning, bone drilling, bone screwing, water jet process, fatigue behavior of implant materials, simulations of COVID-19 and other infections and optimal configuration of implant materials [3–6].

## Methods

### Three-dimensional modeling of the femur and implant

**Fig. 1:**
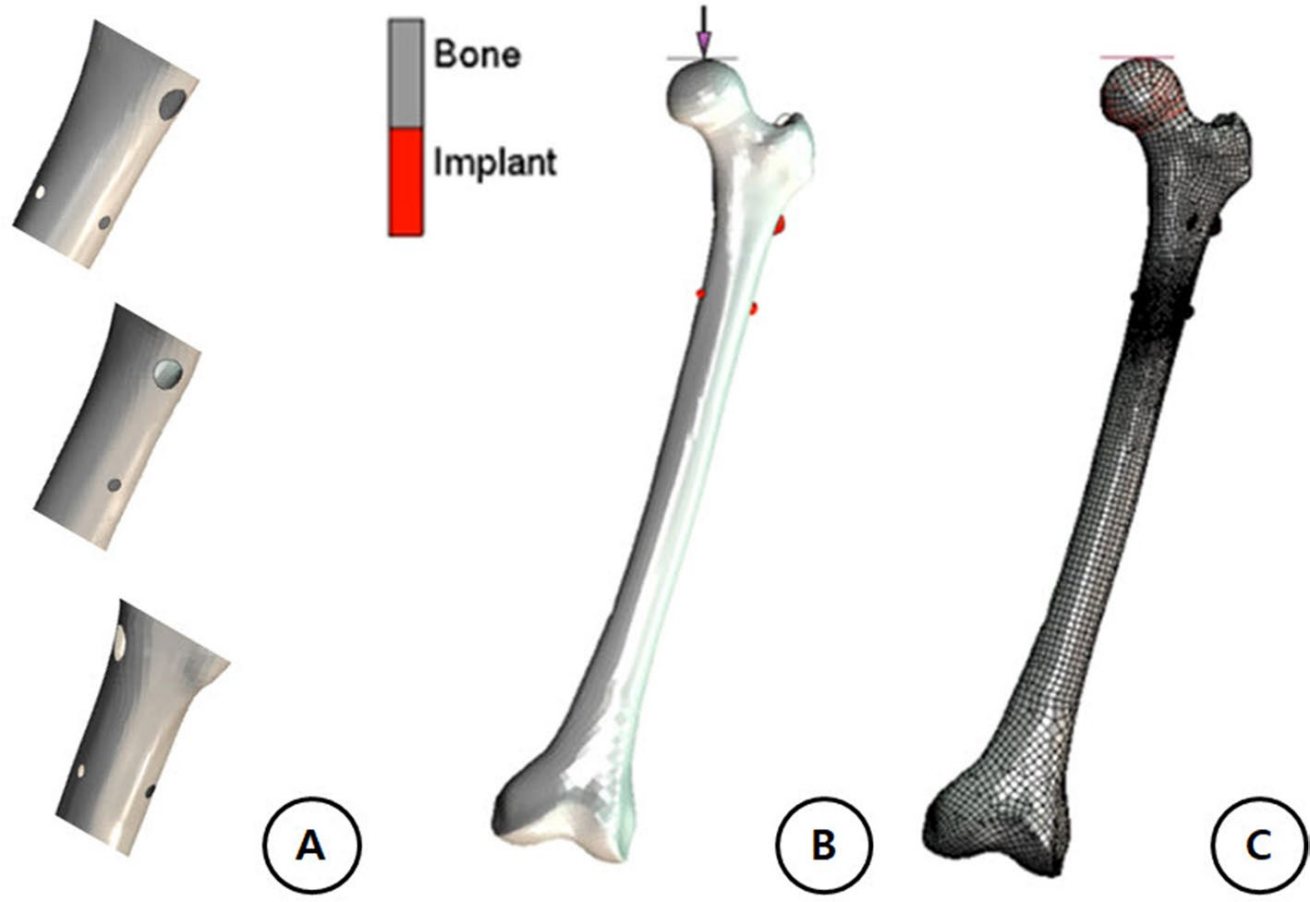
**A** Comparison of stress distribution among three cases according to nail screw hole locations. **B** Creating a rigid body to simulate the experimental conditions of the upper part of the femur, transferring the load to the head of the femur (0–3000 N), and setting the contact glue to the rigid body (cylinder) and the femoral head. **C** The femur is made in three parts of three-dimensional shell modeling, and the Implant (PFNA) is made using Tetra/Hexa Mesh modeling

Computed tomography (CT) scanning with a 1-mm slice thickness was performed on the femur of a healthy, 175-cm tall, 21-year-old man. A solid model of the femur was reconstructed using 3D-DOCTOR software (Able Software Corporation, Lexington, MA) to detect the boundary edge in each slice and Rapid-form-TM software (INUS Technology, Inc., Seoul, Korea) to stack the slices and convert the images into an Initial Graphics Exchange Specification-type model. The dimensions were then keyed into a computer-aided design (CAD) program (CATIA 2016, Dassault, France) to reconstruct the three-dimensional models. The geometrical dimensions of the proximal femoral nail antirotation (PFNA)-II (length of 170 mm, diameter of 10 mm, 5° proximal lateralization angle, helical blade length of 95 mm, caput-collum-diaphyseal angle of 130°) were obtained from the implant manufacturer’s catalog. Then, a geometric model of the implants was assembled as a femur model, and the tip-apex distance was controlled within 20 mm. Assuming that additional holes were formed in the anterior and posterior areas, three models were created by making additional holes with the same diameter as the distal locking screw (transcortical type) through a tangent line of the outer margin and the inner side of the cortex (additional anterior hole [AH], additional posterior hole [PH], and no additional hole [NHO] models) (Fig. 1A). The geometric model of the femur and internal fixation were imported into the FEA pre-processing software Hypermesh 18.0 (Altair, USA) to draw the mesh (Fig. 1B). After the convergence measurement, the mesh size was determined to be 1 mm. FEA was performed using MSC-Marc 18.0 (MSC software, Inc., USA).

### Material properties

All materials were assumed to be linearly elastic, isotropic, and homogeneous [7]. PFNA-II was composed of a titanium alloy. The material properties of the bony models were obtained from the literature [8, 9]. The cortical and cancellous bones were assumed to have elastic moduli of 16.7 GPa and 279 MPa, respectively, and Poisson’s ratios of 0.3 and 0.3, respectively, were used for patients aged 20 years.

### Boundary and loading conditions

A repetitive axial compressive force was applied at the femoral head through a rigid cylindrical body, while the bone was rigidly fixed 20 mm proximal from the distal end of the femur (Fig. 1C). The axial stiffness based on the finite element (FE) computation was 0.54 kN/mm and was within the measurement interval (0.76 ± 0.26 kN/mm) [10]. The individual differences revealed that the FE model was satisfactorily validated. The boundary condition was set by fixing the distal end of the femur, and the displacement along the x-, y-, and z-axes at that site was set to zero [11]. This study simulated the forces acting on the hip during the stance phase of walking [12]. A 3000-N vertical load was applied to the femoral head [13, 14] (Fig. 1B, C). The friction coefficient was 0.3 for each bone-implant interaction [15] and implant-implant interaction [16].

### Observation index

To evaluate the failure of the model, the maximum stress values under von Mises stresses were calculated around the distal locking screw at each load to determine the presence or absence of additional holes and the direction of additional holes. Furthermore, maximum stress values under Comp 11 stresses were calculated to evaluate the possibility of model failure in the tensile or compressive direction. Comp 11 stress was normalized based on a tensile strength of 135 MPa and a compression of 200 MPa.

### Biomechanical experiments

**Fig. 2:**
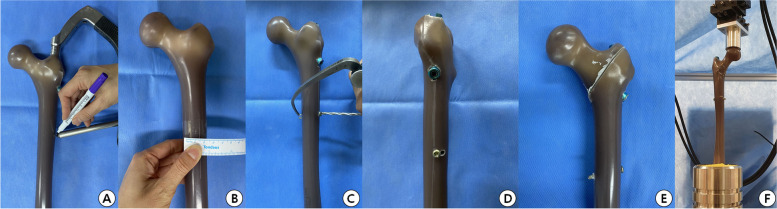
Creating an experimental model of a composite femur bone. **A** Making the start point (SP) using the target device. **B** If the distal locking screw is fixed from the SP and additional holes are formed in the front or rear, the insertion point and exit point are identified. **C** To generate anterior or posterior additional holes or correct hole for distal targeting screw, the holes are drilled using a 4.0-mm drill bite and a 4.9-mm screw is inserted/removed using anterior cruciate ligament reconstruction guide. **D** Model with distal locking screw fixed in the correct position using posterior additional screw hole. **E** All bone model fractures are formed based on Osteosynthesefragen/Orthopaedic Trauma Association classification into trochanteric 31A-21. **F** The femoral head is seated into a customary inverted hemispherical chamber attached to a multidirectional bearing plate fixed to the load cell on the actuator of the material testing machine and then cyclically loaded to failure

This study used a composite bone femur model (Sawbone, Pacific Research Laboratories Inc., WA, USA), an experimental artificial bone widely used in biomechanical studies. The cancellous foam core of the femur model has a density of 17 pounds per cubic foot (density per the Standard Test Method for Apparent Density of Rigid Cellular Plastics (ASTM D1622) was 0.27 with a volume fraction of 0.23), a 12-mm canal, and an overall length of 39.1 cm. The PFN system includes the PFNA-II (nail length 170 cm, diameter 10 cm, helical blade 100 cm) and a distal locking screw with a diameter of 4.9 mm. The PFNA-II, including the distal locking screw (4.9 mm diameter, 38 mm length), was inserted into 30 trochanteric saw bones to form 31A-21 fractures based on the Osteosynthesefragen/Orthopaedic Trauma Association classification. An additional transcortical-type hole (4.9 mm) was made anterior (AH model, 10 cases) or posterior (PH model, 10 cases) to the distal locking screw at the same location (Fig. 2A-E). No additional hole cases (NHO model, 10 cases) were retained for comparison with additional hole cases. All procedures were performed by a single orthopedic surgeon with more than ten years of surgical experience.

### Biomechanical experimental methods

Bone models were mounted at 10° adduction and 11° extension, representing the physiological direction of the maximum applied hip forces during gait, using an MTS 858 Material Testing Machine (MTS Systems Corp., Eden Prairie, MN, USA) [17]. The femoral head was seated in a customary inverted hemispherical chamber attached to a multidirectional bearing plate fixed to the load cell on the actuator of the MTS. The specimens were then cyclically loaded to failure at a frequency of 2 Hz. Cyclic sinusoidal compressive loading with a tapered sine waveform began with a 50–500 N load stepwise, increasing upper loads by 50 N every 2500 cycles, with a maximum applied load of 50–3000 N. Failure was defined as construct breakage around the distal locking screw (Fig. 2F). Fixation loss or displacement of the intertrochanteric fracture was not considered a failure. The peak/valley displacements and loads for each cycle were obtained. The failure cycle (n), maximum load (N), and dynamic stiffness (N/mm) were imputed using these values.

### Statistical analysis

Comparisons between the NHO group and the AH and PH groups were performed using Fisher’s exact test. Survival analysis was performed by considering the cycle information as time and the failure case as the end point. The log-rank test was used to compare survival curves. Statistical significance was defined as a *P*-value < 0.05. All analyses were performed using R 4.0.1 (R Development Core Team; R Foundation for Statistical Computing, Vienna, Austria).

## Results

### FEA of the stress changes in the cortical bone around the additional screw hole

#### von Mises stress distribution

**Fig. 3:**
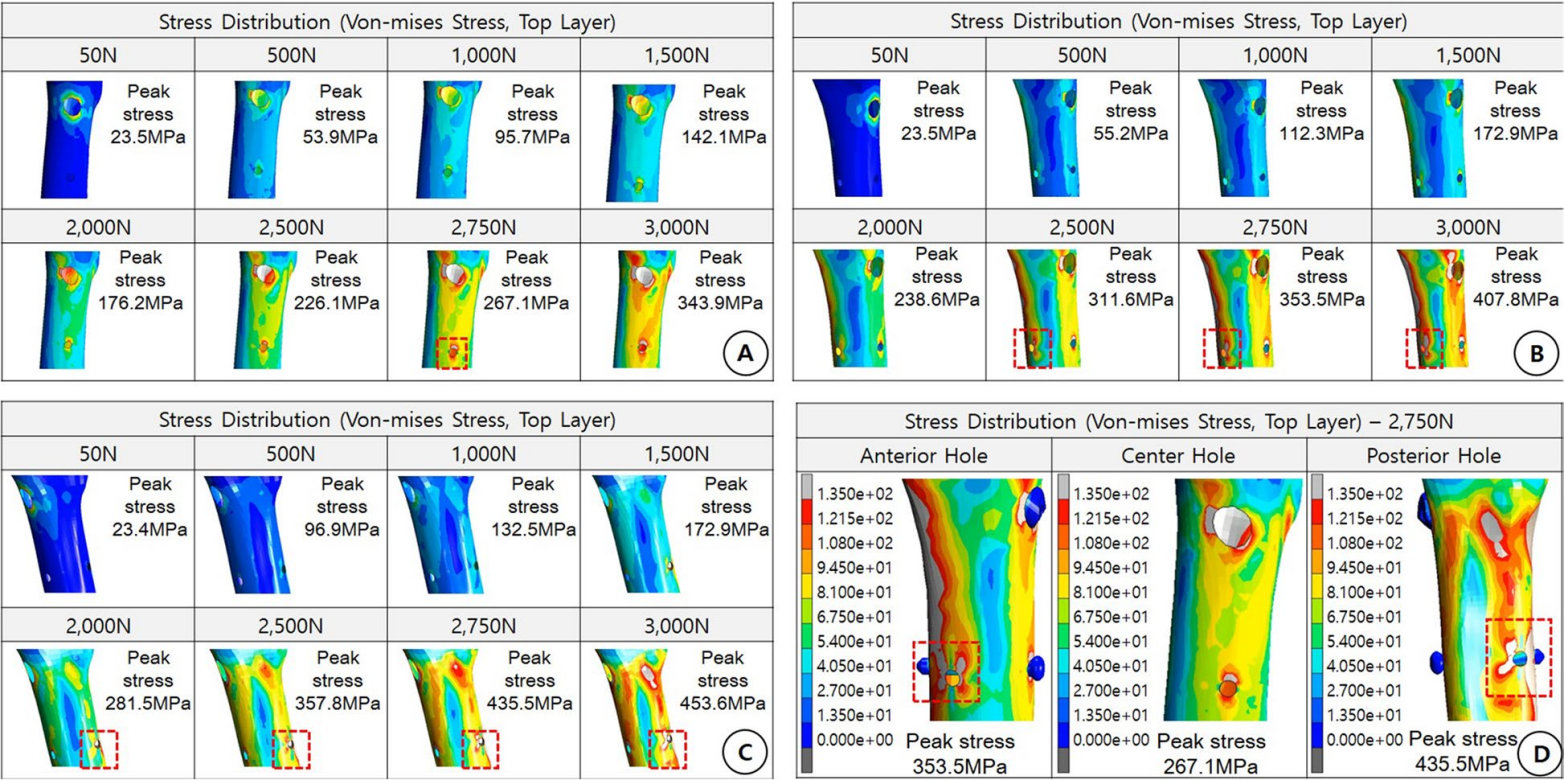
**A** Center hole case. The stress around the hole occurs over the tensile strength (135 MPa) from 2500 N, and local damage may occur because the stress is usually concentrated around the hole. Stress distribution (von Mises stress, top layer). **B** Anterior hole case. Tensile strength (135 MPa) or higher stress occurs from 2000 N of load around the hole. Stress concentration occurs around the anterior additional hole. There is a high possibility of breakage. Stress distribution (von Mises stress, top layer). **C** Posterior hole case. Tensile strength (135 MPa) or higher stress occurs from the load around the hole from 2000 N. Stress concentration around the posterior additional hole occurs. Similar to the anterior case, there is a high probability of breakage. Stress distribution (von Mises stress, top layer). **D** Von mises stress. Tensile strength (135 MPa) or higher stress concentration occurs around the additional holes. This suggests a high possibility of damage by compressive load stress distribution (von Mises stress, top layer) at 2750 N

The von Mises peak stress (226.1 MPa) around the distal locking screw of the NHO model was greater than the compressive strength (200 MPa) from 2500 N, and local breakage might have developed due to local stress concentration around the distal locking screw; however, this was difficult to visualize in cases with complete breakage (Fig. 3A). Nonetheless, the von Mises peak stress (238.6 MPa, 281.5 MPa) around the distal locking screw in the AH (Fig. 3B) and PH (Fig. 3C) models was higher than the compressive strength (200 MPa) from 2000 N; therefore, complete breakage may occur due to local stress concentration around the distal locking screw. The area around the distal locking screw was structurally weaker when there was an additional hole than when there was no additional hole.

This can be explained as follows. The motion direction is attributed to the compressive force, and the stress is concentrated in the additional hole by the compressive load, increasing the possibility of femur fracture (Fig. 3D).

#### Tensile and compressive stress distribution under comp 11 stress

**Fig. 4:**
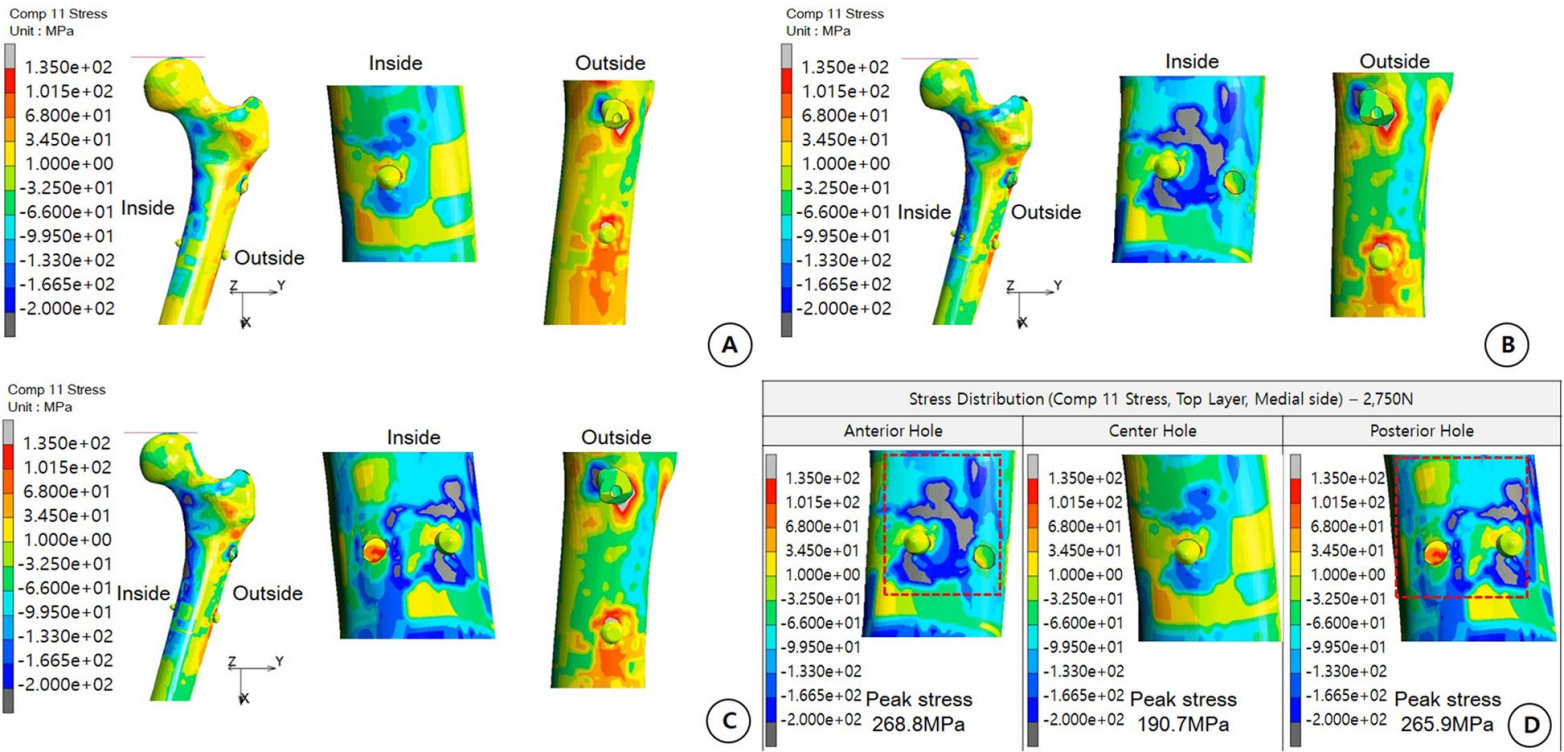
**A** Comp 11 stress (stress in the *x-*axis direction). Stress generation by tensile stress around the medial side of the femur and compressive stress around the lateral side of the femur (compressive stress, 200 MPa). The possibility of damage to the medial side is low and that to the lateral side is also low, except for minute damage around the hole. **B** Comp 11 stress anterior hole case. Under stress distribution, at 2750 N, there is a high possibility of damage around the additional hole due to the medial compression force and lateral tensile force because of structural weakness and a high possibility of damage caused by cracking from the additional hole toward the outer hole. **C** Comp 11 stress posterior hole case. Under stress distribution, at 2750 N, there is a high possibility of damage around the additional hole due to the medial compression force, lateral tensile force, and structural weakness. **D** Center hole case. The possibility of damage is low around the medial side. However, breakage is highly possible when an additional hole occurs. The anterior hole case is the most vulnerable because the maximum stress is higher than in the posterior hole case, and the damaged area is wider

When comparing the medial and lateral sides of the femoral bone in each model under Comp 11 stress (stress in the *x*-axis direction) subjected to the medial compression force of the femoral bone and the lateral tensile force, the maximum stress of the NHO model was 190.7 MPa. Conceivably, the medial side of the NHO model is not expected to be breakage by compressive load, and micro breakage is possible around the distal locking screw on the lateral side; however, the possibility of entire breakage in the femur was thought to be low (Fig. 4A). Therefore, in the AH and PH models, compression on the medial side and tensile force on the lateral side of the femur resulted in a high-stress concentration around the additional hole. Compared to the NHO model, the possibility of femoral fracture around the additional hole was higher, and structural vulnerability was estimated (Fig. 4B, C). In particular, at 2750 N, the maximum stress values of AH and PH on the medial side of the femur were 238.8 MPa and 265 MPa, respectively. The AH model showed a wider distribution of stress above the compressive strength of the femur than the PH model did. These results suggest that the AH model is at a high risk of breakage, making it more vulnerable to fracture than the PH model (Fig. 4D).

#### Load repeat experiment in NHO, AH, and PH models

**Fig. 5:**
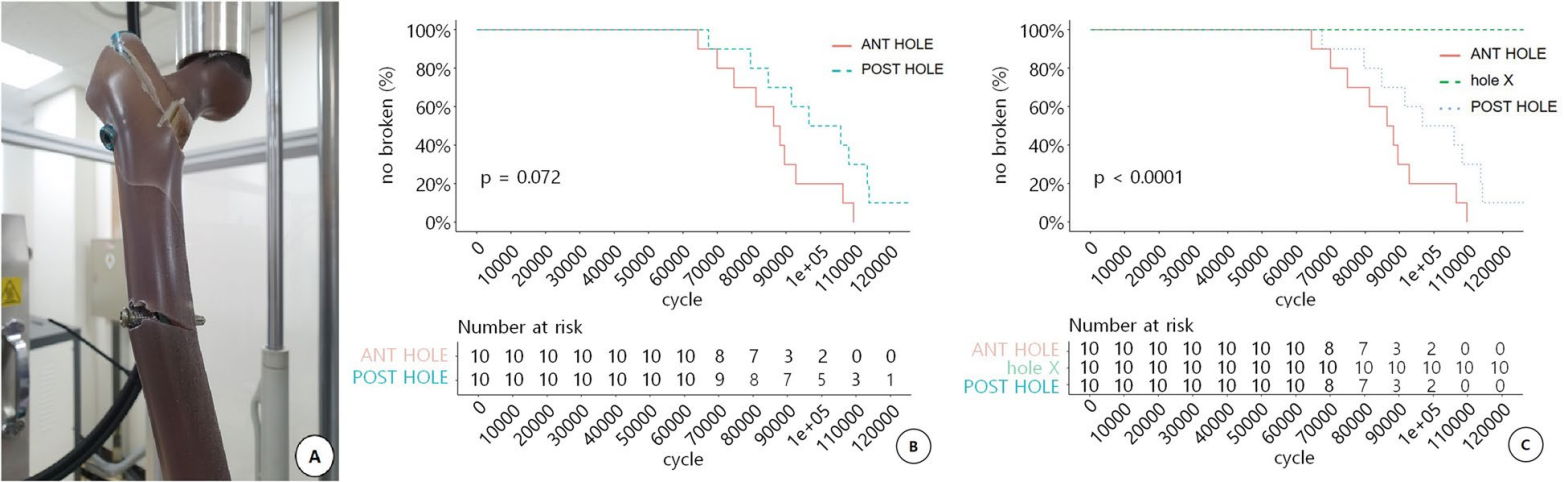
**A** Failure is defined as construct breakage around the distal locking screw, whichever occurred first (**B**) Log-rank test results, which defined cycle value as time and failure as end point, show a significant difference between the NHO group and AH and PH groups (*P* < 0.001). **C** Between AH group and PH group, neither Fisher’s exact test (*P* = 1) nor the Log-rank test (*P* = 0.072) showed a significant difference

Fracture around the distal locking screw, defined as failure (Fig. 5A), was not found in the NHO group, occurred in ten cases in the AH group, and nine cases in the PH group. Among the ten cases of failure in the AH group, the average maximum load was 2195.8 N (1750–2650 N), and the average cycle of load repetition was 86,343.7 (64254–109,500) (Table 1). Among the nine cases of failure in the PH group, the average maximum load was 2363.6 N (1800–2750 N), and the average cycle of load repetition was 94,748.4 (67323–114,012). A significant difference was found in the maximum load and cycle (*P* < 0.001) between the NHO group and the AH and PH groups in Fisher’s exact test. The log-rank test, which defined the cycle value as time and failure as the end point, showed significant differences between the NHO group and the AH and PH groups (*P* < 0.001) (Fig. 5B). Between the AH and PH groups, neither Fisher’s exact test (*P* = 1) nor the log-rank test (*P* = 0.072) showed significant differences (Fig. 5C).

**Table 1:** The results of repetitive-load biomechanical testing

Type (*n* = 10)	Fail	Cycle to fail (min–max)	Newton (N) to fail (min–max)
Model NHO	0	N/A	N/A
Model AH	10	86,343.7 (64254–109,500)	2195.8 (1750–2650)
Model PH	9	94,748.4 (67323–114,012)	2363.6 (1800–2750)

*NHO* no-hole model, *AH* anterior additional hole model, *PH* posterior additional hole model, *N/A* not applicable

## Discussion

Despite the development of implants, devices, and surgical techniques, complications related to the distal locking screw of the PFN system remain [2]. These include insufficient dissection of the fascia, excessive tightening of the distal locking screw, and loosening of the targeting device between the nails [18]. The most common initial complication is unstable coupling. When a distal locking screw is inserted in an incorrect position, it creates an additional hole at the anterior or posterior femur.

The following reasons may explain the possible occurrence of fracture around the hole. First, fractures occur because of cortical bone loss caused by an additional hole. The cortical bone is critical for maintaining the mechanical strength of the bone. In particular, a 3-mm hole in the cortical long bone decreases the bending strength by 40%, while the torsional strength reduces by approximately 12% [19]. However, the PFNA-II uses a 4.9-mm screw, and it is expected that these decreases in bending and torsional strength are even greater. Therefore, the authors estimated that repeated loads might induce fractures when such a large amount of cortical bone loss negatively affects the mechanical strength of the femur.

Second, the type of additional holes may affect the incidence of fracture. Additional screw holes can be classified into unicortical, bicortical, half-bicortical, and transcortical penetrations [20]. Most additional screw holes that are directed anteriorly and posteriorly to avoid nails are of the transcortical type. It is supposed that the highest stresses result from transcortical penetration, and the fracture risk ratio was dramatically elevated under both axial and torsional loads [21]. If an additional screw hole occurs in a patient with a large proportion of the nail in the medullary cavity, such as in an Asian with a small body frame, the hole follows a transcortical shape and is pushed outward from the center by the nail, which can generate a risk condition for fracture. In addition, thermal bone necrosis that occurs during transcortical penetration may further increase fracture risk [22].

Third, the stress concentration around the additional holes may influence the occurrence of fractures. The standard walking condition results in the bending of the bone during the stance phase. This generates tensile stress in the lateral femur [23]. The peak stress concentration of the femurs in trochanteric fractures was mainly located in the site where the distal lock screw was making contact [24]. Robinson et al. [25] suggested that cortical bone hypertrophy around the distal nail, observed on a simple radiograph during the healing process of intertrochanteric fractures using an intramedullary nail, was a radioactive hallmark, suggesting that stress concentration occurred around the distal nail [26]. Stress concentration can reduce the mechanical integrity of a bone, making it more susceptible to sudden brittle fracture during trauma or to gradual fatigue failure over time (stress fractures). A distally locked construct bears most of the load, which is gradually transferred to the distal cortex as the fracture heals. With good cortical apposition of the fracture, the bone cortices support most of the compressive load. Without cortical contact of the fracture, the entire load is transferred to the distal screw through the nail until the fracture heals [26]. Therefore, efforts should be made to increase the stable cortical bone contact surface of the fracture through possible anatomical reduction before nail insertion.

Fourth, the mechanical influence should be considered. Additional holes allow the movement of the nail at its junction in the medulla. This movement may cause the nail to slip anteriorly or posteriorly and lead to the eccentric position of the nail in the medullary canal, which will direct mechanical stimulation to the anterior or posterior side of the femur and may cause a fracture [27, 28]. In particular, Asian women have shorter femoral necks and smaller femoral neck angles and increased anterior bowing of the shaft than Western women do. Therefore, the nail could be located eccentrically in the intramedullary space [29], leading to higher fracture risk. Notably, biomechanical loading tests and FEA studies have shown that AH was more susceptible to fracture than PH. The femur with anterior bowing is subjected to increased posteromedial compressive force and anterolateral tensile force. Considering its physical properties, which are more vulnerable to tensile strength, the bone may be more susceptible to fracture when an AH is formed. Therefore, if an AH occurs in older patients with osteoporosis and severe bowing, the risk of fracture would be further increased.

This study had some limitations. First, this study did not consider various forms of load from the human body and did not use real human bones. The authors used a composite femoral bone model to overcome this problem, determined ten identical conditions for each experimental condition, investigated the stress change according to the load using FEA, and further investigated the effect of bone density. Second, the study did not reflect various fracture types and reduction statuses. The authors applied a fracture model with the highest severity among fracture types, excluding reverse oblique intertrochanteric fracture, for which the prognosis is known to be poor. The experiment was conducted assuming that stable reduction was obtained. Third, various types of additional holes, such as unicortical, bicortical, half-bicortical, and transcortical penetrations, were not tested. However, the authors chose the transcortical type to focus on in this study because this type is the most common and the most susceptible to fracture. Finally, the effects of the various diameters of the medulla and nail and the position of the distal end of the intramedullary nail were not considered, and various designs of the PFN system were not tested. Future biomechanical studies incorporating many cadaveric bone models under various conditions and nail systems are recommended.

## Conclusion

During surgery for intertrochanteric fracture using the PFN system, when the distal locking screw is malpositioned anterior and posterior to the nail, the remaining hole may be a risk factor for fractures around it once removed. Therefore, the surgeon should pay great attention during the operation to prevent the accidental occurrence of additional screw holes.

## Data Availability

The datasets during and/or analysed during the current study available from the corresponding author on reasonable request.

## Abbreviations

AH: Anterior hole
CT: Computed tomography
CAD: Computer-aided design
FEA: Finite element analysis
MTS: Material Testing Machine
NHO: No additional hole
PH: Posterior hole
PFN: Proximal femoral nail
PFNA: Proximal femoral nail antirotation
